# Supramolecular chemistry in lipid bilayer membranes

**DOI:** 10.1039/d1sc03545b

**Published:** 2021-07-28

**Authors:** Laura E. Bickerton, Toby G. Johnson, Aidan Kerckhoffs, Matthew J. Langton

**Affiliations:** Department of Chemistry, University of Oxford Chemistry Research Laboratory 12 Mansfield Road Oxford OX1 3TA UK matthew.langton@chem.ox.ac.uk

## Abstract

Lipid bilayer membranes form compartments requisite for life. Interfacing supramolecular systems, including receptors, catalysts, signal transducers and ion transporters, enables the function of the membrane to be controlled in artificial and living cellular compartments. In this perspective, we take stock of the current state of the art of this rapidly expanding field, and discuss prospects for the future in both fundamental science and applications in biology and medicine.

## Abstract 2

This perspective provides an overview of the current state of the art in supramolecular chemistry in lipid bilayer membranes, including receptors, signal transducers, catalysts and transporters, and highlights prospects for the future.

## Graphical abstract

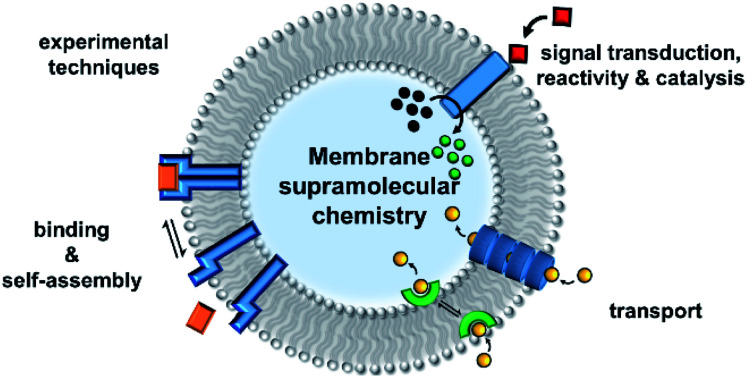

## Introduction

Supramolecular chemistry is the broad area of molecular sciences concerning the study and application of intermolecular interactions in order to develop functional molecular systems. These include new materials, complex molecular topologies, molecular machines, receptors and sensors, amongst many others. Membrane supramolecular chemistry aims to exploit these concepts to develop membrane-bound analogues that enable the functions of the membrane to be controlled in some way.

Lipid bilayer membranes are essential components of living systems, forming compartments that physically decouple the chemical environment on either side of the bilayer. Integrated and peripheral membrane proteins provide additional functionality; controlling molecular recognition and anchoring processes, transmembrane transport of ions and small molecules, and signal transduction and amplification. Such proteins are typically stimuli-responsive; regulating their activity in response to environmental changes. These biological functions have motivated supramolecular chemists to design artificial systems inspired by the functions of these proteins. Key applications of membrane-bound supramolecular systems include ion transport, signalling and molecular sensing. Early work in membrane supramolecular chemistry was motivated by scientific curiosity, and a desire to understand biological mechanisms. This is still a key driving force in the now thriving field, but we are also beginning to see application driven research, particularly in the area of ionophore therapeutics.

A major advantage for a supramolecular chemist working with membranes is that by incorporating organic supramolecular components into hydrophobic bilayers, the chemist has at their disposal a versatile method of interfacing lipophilic supramolecular components with aqueous, biological environments. This is particularly useful because the vast majority of functional organic supramolecular systems are not water soluble. Because of this, it is reasonable to assume that membrane chemistry is likely to be an important area in which we can expect to see useful applications of supramolecular chemistry through its impact on biology and medicine.

**Fig. 1:**
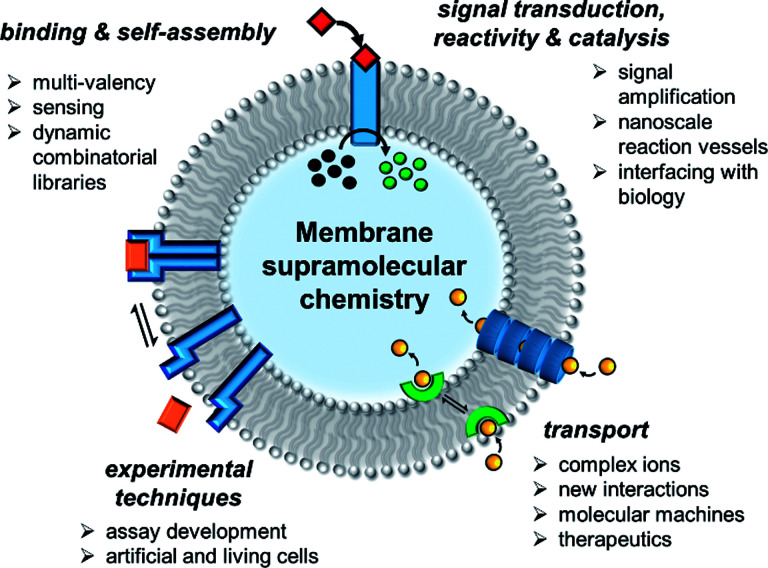
State of the art in supramolecular chemistry in lipid bilayer membranes and future perspectives.

In the past decade there has been a dramatic expansion of the field, and numerous key advances have been published very recently. In this perspective, we take stock of the current state of the art in membrane supramolecular chemistry, and look forward to where the field may be heading in years to come. We highlight key current capabilities in membrane-confined receptors and sensors, signal transduction, catalysis and transmembrane transporters, with the primary focus on developments from the past 5 years (Fig. 1). We conclude by speculating on future challenges, opportunities and applications.

## Molecular recognition and sensing at a bilayer interface using membrane-bound receptors

### Experimental techniques

**Fig. 2:**
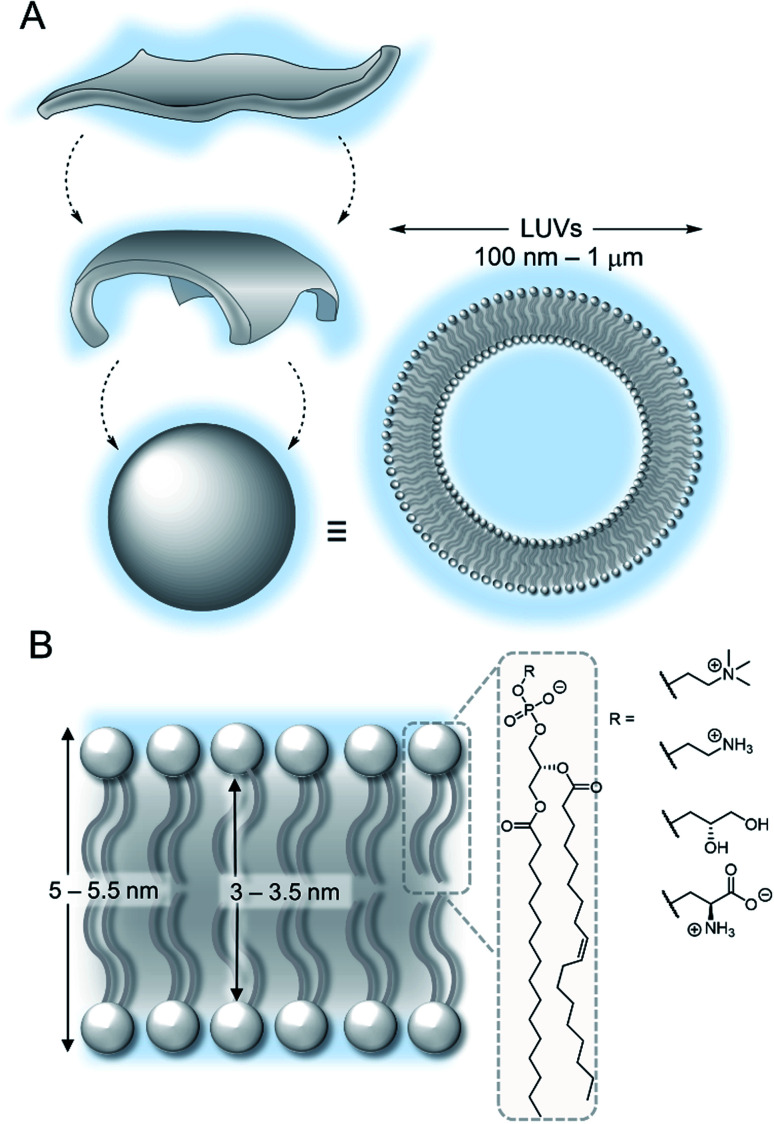
(A) Self-assembly of lipids into vesicles (B) structure of a lipid bilayer membrane and phospholipids.

The lipid bilayer of the plasma membrane functions as a hydrophobic barrier towards polar molecules and is essential for cellular compartmentalisation.^1^ Membranes in nature are predominantly composed of phospholipids. Self-assembly of these amphiphilic phospholipids occurs under aqueous conditions to form two-dimensional bilayers, which spontaneously form sealed compartments to minimise interactions of the hydrophobic fatty acid chains with the aqueous environment (Fig. 2A). These bilayers form the basis of cellular membranes and liposomes.^1^ The phospholipids are typically formed from two hydrophobic fatty chains linked *via* ester bonds to glycerol (Fig. 2B). This is in turn is attached to choline or ethanolamine *via* a phosphate ester bond to form zwitterionic lipids; or to serine or glycerol derivatives to form anionic lipids. The thickness of the hydrophobic interior of the membrane is typically 3–3.5 nm, depending on the composition, with an overall membrane thickness of around 5–5.5 nm including the hydrophilic phosphate head groups.^2^

Synthetic membranes can be conveniently generated from commercially available phospholipids, and the formation of lipid bilayer vesicles with a high level of control over size and contents is relatively straightforward.^3^ Large unilamellar vesicles (LUVs, 100 nm to 1 μm in diameter) containing fluorescent probes are routinely used to study supramolecular systems, because of their stability and ease of preparation *via* extrusion methods.^4^ Giant unilamellar vesicles (GUVs, diameter >1 μm) offer the benefit that individual vesicles are directly observable by microscopy but are typically less stable than LUVs.^5^ Ion channels are often also studied using planar lipid bilayer techniques, in which a bilayer is formed across a micrometre scale aperture that separates chambers filled with aqueous solutions. Single channel electrical recordings of the ion current through a membrane channel is used to characterise the ion transport capability of the system.^6^ More recently, amphiphilic block copolymers have been used to form vesicles (polymersomes), which benefit from improved stability against dilution and enhanced chemical diversity.^7^ A detailed discussion of the various experimental techniques available is beyond the scope of this perspective, and we direct the reader to a number of review articles on the subject.^3,5^

### Molecular recognition and sensing at a bilayer interface using membrane-bound receptors

Confinement of supramolecular receptors within a lipid bilayer membrane leads to an increase in effective concentration by many orders of magnitude, because the receptors are confined to the volume of the membrane. For example, in a typical fluorescence ion transport assay, a total concentration of receptor (ion carrier) of 100 nM in vesicles with lipid concentration of 100 μM experiences an effective concentration in the membrane of ∼1 mM, because the membrane-embedded carrier occupies only a small volume fraction of the solution that comprises the lipid membrane. Furthermore, the strength of intermolecular interactions are enhanced relative to those in aqueous solution because of the lower polarity of the membrane environment. These factors can lead to different binding processes at membrane interfaces to those observed in solution, such as the formation of high receptor to guest stoichiometry, multivalency,^8–10^ and receptor clustering effects.^11,12^ These effects have been exploited by supramolecular chemists to develop a wide range of recognition and sensory systems.^13,14^

**Fig. 3:**
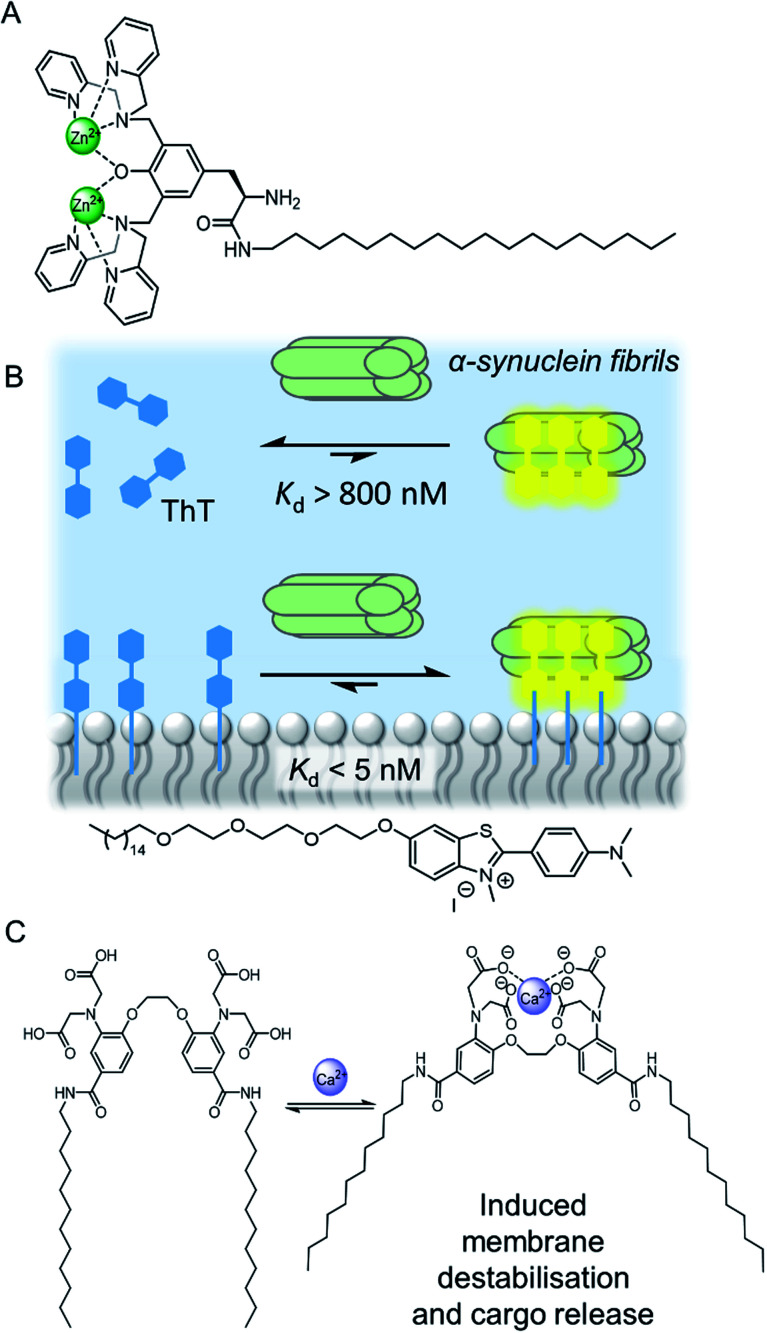
(A) Membrane anchored zinc complex for peptide recognition.^15^ (B) Binding of amyloid protein aggregates enhanced by membrane anchoring of fluorescent thioflavin-T (ThT).^16^ (C) A calcium responsive lipid switch.^17^

Arguably a key benefit of anchoring receptors within a fluid membrane rather than, say, covalently attached to a surface, is the ability for receptors to reorganise in order to facilitate target binding. This effect has been utilised to develop a variety of metal ion sensors, by co-embedding receptors and fluorescent reporter groups within a bilayer. For example, König and co-workers utilised membrane fluidity to assemble a recognition platform for peptides.^15^ High affinity nanomolar binding of a model peptide was achieved in fluid DOPC membranes using membrane-anchored bimetallic complexes such as the zinc complex in Fig. 3A, for recognition of *O*-phosphoserine (pSer) and histidine (His) moieties in the peptide. In contrast, binding was two orders of magnitude weaker in saturated DSPC lipid membranes, where low lateral mobility in the gel phase membrane inhibits dynamic receptor reorganisation.

Co-localisation of fluorescent molecules and supramolecular receptors enables membrane binding of analytes to be detected *via* an optical response. The König group, for instance, have used their membrane-confined zinc complexes to develop sensors for a range of biomolecules, including phosphorylated peptides,^18^ enzymes,^19^ and the products of enzymatic reactions.^20^ Recently Hunter and co-workers have used vesicle-mounted fluorescent dyes to sense α-synuclein fibrils.^16^ Using vesicle-mounted thioflavin-T (ThT), a high contrast fluorescent probe used to image amyloid aggregates that are implicated in degenerative diseases such as Alzheimer's, nanomolar affinity (*K*_d_ < 5 nM) for amyloids with enhanced brightness from the probe was achieved (Fig. 3B). In contrast, monomeric ThT in solution binds two orders of magnitude more weakly.

Combining a receptor with a conformational switch can be used to control release of cargo from inside liposomes.^21^ Best and co-workers have reported a calcium-responsive lipid switch which destabilises the membrane upon metal binding by driving the divergence of the appended alkyl chains. This leads to the release of the entrapped dye sulforhodamine B (Fig. 3C).^17^ A similar concept has been reported very recently by Pedersen and co-workers, who showed that conformational changes in a carbohydrate derived lipid in response to Zn^2+^ binding triggered cargo release from vesicles.^22^

The examples above concern intra-vesicle molecular recognition, but synthetic receptors can also be used to mediate inter-vesicle interactions *via* hydrogen bonding or metal–ligand interactions, which lead to vesicle adhesion and in some cases, fusion (in a similar manner to SNARE proteins in biology).^23^

## Supramolecular control over reactivity in membrane-bound compartments

### Membrane-anchored catalysts

The nanoscale compartment of lipid bilayer vesicles offers a unique environment in which to control chemistry. The membrane separates the aqueous interior from the exterior, which may be chemically different, whilst providing a hydrophobic medium in which reagents, products and catalysts can be localised.^24^ The high effective concentration and promotion of intermolecular interactions within the bilayer is particularly advantageous for enhancing reaction kinetics. However, the reactivity of species trapped in the aqueous solution inside a vesicle can also be different to those in the exterior solution, even if the species are not confined to the membrane, because in small compartments these species experience a large local lipid concentration.^25^ The control of reactions in artificial vesicles and living cells, such as using transition metal catalysts, is a large and growing field that is beyond the scope of this perspective. Readers are directed to recent reviews.^24,26–30^ Here we focus on selected recent examples to highlight the concepts from a supramolecular viewpoint.

**Fig. 4:**
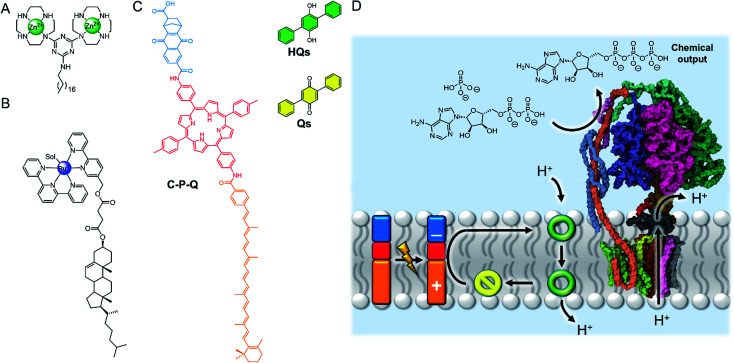
(A) Membrane-anchored zinc catalyst.^31^ (B) Lipophilic ruthenium complex for O_2_ evolution.^34^ (C) Photo-driven active transport of protons by photo-induced electron transfer from the membrane-embedded carotene–porphyrin–naphthoquinone (C–P–Q) to the redox-switchable quinone ion carrier Qs.^35^ (D) Dissipation of the proton gradient through ATP synthase (PDB ID: 5FIL) leads to the chemical synthesis of ATP from ADP.^36^

The high effective concentration of membrane-anchored species has been exploited by König and co-workers to develop vesicles decorated with pendant zinc complexes able to promote hydrolytic DNA cleavage (Fig. 4A).^31^ The membrane anchored catalysts aggregate within the bilayer, leading to enhanced reactivity through high local concentrations. The decreased polarity at the bilayer-aqueous interface further promotes catalytic activity. Catalytic activity of membrane-bound complexes can also be tuned by co-localising additives within the membrane,^32^ including enantioselective hydrolysis reactions where chiral amphiphiles are included in the membrane.^33^ The fluidity of the membrane scaffold was found to be a significant factor affecting catalysis rates; the fluid membrane of DOPC favoured catalysis, while the gel phase of DSPC limited mobility of the membrane bound additives and reduced catalytic activity and enantioselectivity.

### Artificial photosynthesis

The ability of the membrane to maintain charge-separated states by preventing ion permeation is essential for photosynthesis. There has been a drive to harness photo-induced charge separation in artificial cells by incorporating biological light-driven proton pumps, such as bacteriorhodopsin, in order to generate an ion concentration gradient across the membrane.^34,35^ Initial progress in developing entirely artificial systems has focused on using photosensitising coordination complexes immobilised within the membrane to promote photo-catalyst-mediated reactions.^36–39^ For example, the membrane confinement of catalytic ruthenium complexes enables highly efficient photocatalytic water oxidation, at much lower concentrations of catalyst than in homogeneous solution.^36^ The highest turnover numbers (TONs) were obtained in gel phase lipids, suggesting that clustering and limited dynamic mobility enhances photocatalytic activity. The nature of the lipid head group also affects the catalytic activity of membrane-bound metal complexes. Ohba and co-workers showed that the activity of a membrane-anchored ruthenium catalyst (Fig. 4B), able to regulate O_2_ evolution in the presence of a cerium(iv) ammonium nitrate oxidizing agent, was dependent on the charge of the lipid head group; altering the catalyst–cerium(iv) interaction and hence electron transfer process.^40^

Photo-induced charge separation across an artificial membrane has been utilised for the active transport of ions.^41,43,44^ For example, Gust and co-workers developed an artificial photosynthetic system (C–P–Q) comprised of an electron donor carotenoid and an electron acceptor quinone coupled through a photo-chemically active porphyrin (Fig. 4C).^41^ Since this species is asymmetric, it preferentially embeds into the bilayer carotenoid end first. Photo-irradiation forms a stable charge separated species, generating a redox potential difference across the bilayer that can be used to drive electrochemical active transport of protons in the presence of a protonophore.^41^ Incorporating ATP synthase into the membrane led to the generation of ATP *via* dissipation of the proton gradient, demonstrating the state of the art in harnessing photochemical energy for the synthesis of complex substrates (Fig. 4D).^42^ Matile and co-workers have also reported photo-induced charge separation in a rigid-rod π-stacking channel structure, which drives a transmembrane ion gradient.^44^

### Dynamic combinatorial chemistry in lipid bilayer vesicles

Lipid bilayer vesicles have been used as a platform for modulating the properties of dynamic combinatorial libraries (DCLs). In a DCL, reversible covalent bonding enables the interchange of different chemical components in a mixture. The composition of this mixture, which is under thermodynamic control, can be modulated by a template to amplify a complementary target.^45^ In the presence of lipid bilayer vesicles, DCLs may be biased in favour of one product over another. This is driven either by differing membrane partitioning of library members, as demonstrated by Bravin and Hunter,^46^ or the high effective concentration of membrane-confined components. An example of the latter was reported by Otto and co-workers, who demonstrated that the composition of a DCL, built up of thioester components that can undergo rapid thioester exchange reactions, is biased towards larger linear species.^47^ In contrast, analogous DCLs in bulk aqueous solution comprises higher populations of small macrocyclic species. This is attributed to the high effective concentration of the DCL building blocks in the membrane, which shifts the equilibrium in favour of intermolecular covalent bond formation to form chain-like species. Dynamic bond formation can also occur between vesicles, as shown by Ravoo and co-workers who have exploited dynamic covalent reactions to control reversible inter-vesicle cross-linking and aggregation.^48^

## Transmembrane signal transduction and catalysis

Signal transduction involves the transfer of information across the bilayer, rather than the physical exchange of matter. In nature, cellular signalling mediated by membrane spanning proteins is highly specialised to respond to the numerous stimuli in the cell's local environment, such as small signalling molecules (primary messengers), membrane potential and light. There are two mechanisms by which signal transduction occurs; namely a conformational change of a membrane spanning protein induced by the input signal, and dimerization of membrane spanning receptors. Inside the cell, the signal is typically propagated through release of a secondary messenger molecule. Amplification of low concentrations of primary messenger is achieved by the activation of catalytic processes. Overall, the process can be summarised by three key steps: stimulation by the primary messenger; signal transmission across the bilayer without mass transport; and amplification with release of the second messenger. The complexity of the process has somewhat hindered the development of artificial signalling systems, and systems that can couple all three steps of the process together are particularly rare.^49^

### Signal transduction mechanisms mimicking nature

**Fig. 5:**
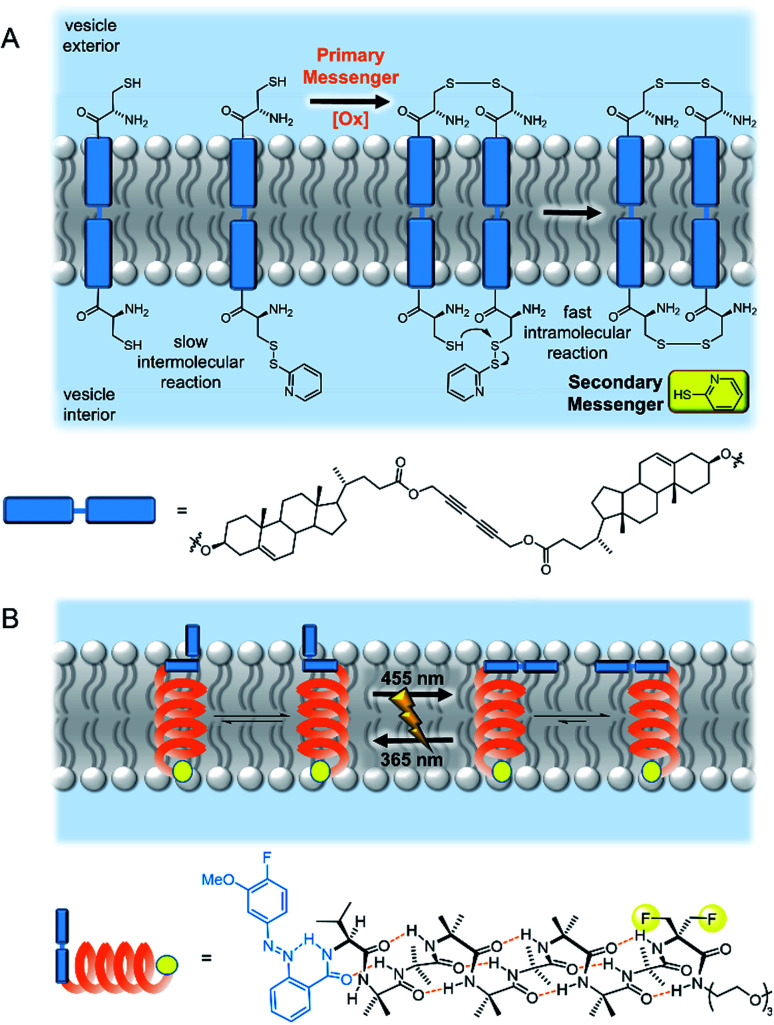
Artificial transmembrane signal transducers mimicking natural mechanisms. (A) Receptor dimerisation^50^ and (B) conformational change.^55^

The biotic mechanism of dimerization of membrane spanning receptors was the first to be addressed in a synthetic system. Hunter, Williams and co-workers demonstrated that oxidative disulfide coupling of two membrane spanning signalling molecules at the outer leaflet brought their reactive terminals together at the inner leaflet, leading to a fast intramolecular disulfide exchange reaction with release of the second messenger, pyridine-2-thiol (Fig. 5A).^50^ The cooperative effects of pre-organisation in the bilayer were further exemplified in a later study in which enhanced guest binding at the inner leaflet was observed by dimers assembled through binding a primary messenger at the outer leaflet.^51^ Schrader and co-workers later reported related systems in which recognition of a primary messenger (diethylenetriamine) by bis-phosphonate recognition groups induced dimerization of the membrane-spanning species, resulting in either a fluorescent output due to FRET between donor and acceptor labelled signalling molecules,^52^ or release of the same pyridine-2-thiol secondary messenger.^53^ The application of these artificial signalling systems for use in controlling reactivity within vesicles is hindered by the significant background release of secondary messenger from intermolecular reactions of the components in the bilayer.^53^

A similar dimerization approach has recently been reported, in which ATP triggers the dimerization of artificial DNA-based receptors. These receptors are coupled to the G-quadruplex-mediated peroxidation of Amplex Red, leading to the generation of resorufin and fluorescence output within vesicles.^54^

The second biological mechanism of signal transduction, namely conformational changes of membrane-embedded receptors, has been explored by Clayden and co-workers as a means of information transfer along foldamers embedded in a bilayer.^55,56^ Foldamers have been proven to be effective signalling molecules that operate *via* a conformational change mechanism, because of their efficient and predictable global molecular reorganisation over the large distance required to propagate a signal through the 3–5 nm thick bilayer.^57^ Clayden and co-workers initially developed a photo-responsive artificial signalling system which displayed photo-regulated conformational change within a bilayer membrane (Fig. 5B).^55^ Subsequent work expanded the concept to include a related system with a carboxylate binding site, in which a conformational change is triggered in response to binding of a chiral carboxylate. The helical screw sense of the foldamer could be controlled by the chirality of the primary messenger, and was reported by fluorescence from pyrene fluorophores at the other end of the foldamer.^56^

Converting this signal propagation process into a full signal transduction system that leads to a chemical intra-vesicle/cellular response has yet to be demonstrated. This is likely to be due to the difficulty in developing signal transducers which display binary switching between inactive (OFF) and active (ON) states. The systems discussed here display a population bias for a certain conformation in response to a signal, but there remains a significant population of the alternate conformation, which if coupled to a downstream catalytic process would be expected to lead to a background response in the “OFF” state.

### Abiotic mechanisms for signal transduction

**Fig. 6:**
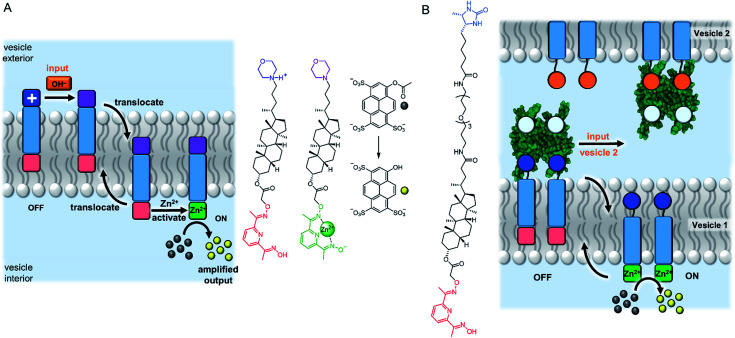
Signal transduction and amplification *via* translocation. (A) pH-controlled system.^58^ (B) Inter-vesicle communication mediated by binding of desthiobiotin-functionalised transducers to NeutrAvidin (PDB ID: 4I60).^59^

An entirely abiotic mechanism of signal transduction has been developed by Hunter, Williams and co-workers, which addresses the challenge of switching reversibly between ON and OFF states with limited background activity. This mechanism is based on the controlled translocation of membrane embedded synthetic signalling molecules from one side of the vesicle membrane to the other.^58–62^ Switching of an anchoring head group between a charged (blue) and neutral (purple) state in response to an external stimulus (orange) facilitates control over membrane translocation of the signal transducer (Fig. 6A). Zinc-binding to a pyridine oxime catalytic head group (red to green) on the other end of the molecule provides a means to amplify and propagate the signal inside the vesicle. Numerous artificial primary messengers have been employed to control signal transduction, using head groups which respond to pH change (Fig. 6A),^58^ metal complexation^60^ and redox switching.^62^ The catalytic head group catalyses the hydrolysis of an encapsulated ester substrate, producing either an amplified fluorescent response, or generating a surfactant to trigger the release of a co-encapsulated dye.^61^ In the absence of the input signal, the catalytic head group is not complexed to zinc because it is held inside the bilayer, which leads to minimal background activity caused by the signalling system itself. Recently the same team elegantly showed that such an abiotic signalling system could be controlled using biotic protein-based binding motifs.^59^ In this variation, a NeutrAvidin protein bound to desthiobiotin headgroups of two signal transducers form the OFF state (Fig. 6B). While biotin has a markedly greater binding affinity for NeutrAvidin compared to desthiobiotin in bulk aqueous solution, it was found that the multivalent binding of membrane-anchored desthiobiotin was too strong to be displaced by the addition of free biotin ligands. Displacement of the NeutrAvidin protein and initiation of the signal transduction mechanism could be achieved using another population of vesicles decorated with membrane-anchored biotin, to take advantage of the enhanced multivalent binding effect.

**Fig. 7:**
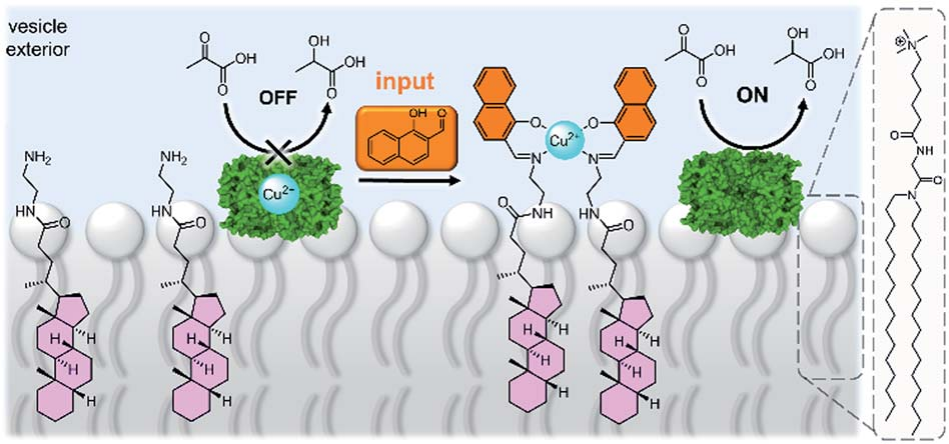
Supramolecular control over lactate dehydrogenase (PDB ID: 5LDH) activity on the surface of vesicles.^63^

A system that achieved integration of membrane bound supramolecular signal transducers with enzymes was developed by Kikuchi and co-workers.^63^ The system utilises vesicles prepared from synthetic cationic lipids and lactate dehydrogenase (LDH) enzymes inhibited by bound Cu(ii) (Fig. 7). Addition of the signal (an aldehyde) generates a membrane bound imine receptor for Cu(ii) ions, which in turn switches on the enzyme activity. Cu(ii) ion receptors which are responsive to competitive guest binding,^64^ chemical signal adduct formation^65,66^ and light^67,68^ have also been utilised. In these examples, the enzyme and signalling molecule are on the same side of the membrane, and without physically decoupling the signal from the effect the membrane serves only to localise components on the vesicle exterior.^69^ As far as we are aware, no synthetic transducers have been reported that exploit the compartmentalisation afforded by the lipid bilayer to regulate enzyme activity.

## Ion transport systems

In nature, membrane-spanning proteins mediate the selective transport of ions across the lipid bilayer by providing a hydrophilic pathway for the ion. Using protein channels in research applications can be limited by availability, scale and cost of the proteins;^70^ or lack of desired function if abiotic applications are desired.

The development of synthetic transport systems for both ions and water has now developed into a well-established field in its own right. Synthetic transporters are attractive for manipulating ion transport in artificial or living cells, and show promise in the treatment of diseases. Many examples act as antibacterial and anti-cancer agents, or as potential therapeutics for diseases arising from mis-regulated ion channels, which are known as channelopathies. Synthetic ion transporters may be classed as either membrane spanning pores/channels, or mobile carrier systems. The latter are lipid soluble ion receptors that bind the ion at the interface, diffuse across the membrane as the ion–transporter complex, and release the ion on the other side. A comprehensive discussion of this field is beyond the scope of this perspective, and so we choose to highlight recent advances and the state of the art in this field. For further reading, we direct the reader to recent review articles.^71–76^

### Synthetic ion channels

#### Self-assembled cages and MOFs

Recently, organic and metal–organic cages have emerged as a viable strategy towards achieving transmembrane ion and water transport. Cages benefit from the ability to control the size of well-defined pores, cavities and windows, often leading to high selectivity towards specific ions, and can additionally be engineered to span the entire lipid bilayer. Moreover, they are frequently prepared from relatively simple building blocks and modified in a modular fashion to optimise properties such as lipophilicity, size or solubility. The cages are generally assembled externally and are added to the membrane as the pre-formed construct,^77^ but can also be generated within the lipid bilayer from sub-components.^78^

**Fig. 8:**
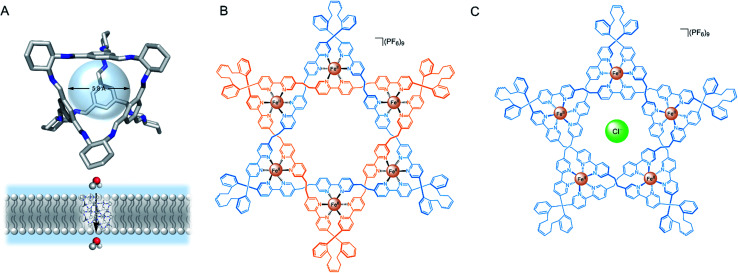
(A) Solid state structure and schematic of porous organic cage for transmembrane water transport.^80^ (B) Leigh's Star of David catenane and (C) pentafoil knot.^81^

Several impressive examples of cage-based ion channels have recently been reported. Kim and co-workers developed a stable 3-D porphyrin-derived organic cage which spans the width of a lipid bilayer.^77^ The windows and internal cavity of the cage facilitate selective iodide transport when embedded within both lipid bilayer vesicles and kidney cells. Nitschke, Keyser and co-workers prepared a stable membrane-spanning Zn_10_L_15_ metal–organic cage *via* sub-component self-assembly, which is capable of transporting anions across the membrane of both planar lipid bilayers and LUVs.^79^ The authors observed that the system acts as a ligand-gated ion channel, in which dodecyl sulfate binding inhibits ion transport. Zhao and Jiang prepared a series of highly symmetrical tetrahedral porous organic cages capable of efficiently transporting water across lipid bilayers with high ion rejection (Fig. 8A).^80^ The authors postulate that the ∼2 nm cages form highly ordered crystalline nanoaggregates that span the lipid bilayer. Water transport rates approaching that of natural aquaporins were achieved, especially with cages exhibiting larger window sizes, higher structural rigidity and appropriately balanced hydrophilicity within the cavity (*i.e.* hydrophilic enough to attract and store water but not bind it too strongly). The authors reason that the ions were impermeable in systems with larger window sizes due to the repeated dehydration penalties required to pass through the extended network of cages. The smaller, hydrophobic cages exhibiting pore sizes <7 Å are also impermeable to ions because they are unable to compensate for the ion dehydration penalty with favourable interactions.

More recently, Leigh and co-workers demonstrated that a Fe(ii) 6-coordinated “Star of David” catenane formed a unimolecular anion channel (Fig. 8B).^81^ Fe(ii) cations were necessary for activity, which the authors attribute to increased rigidity and improved anion binding. The closely related pentafoil knot (Fig. 8C) was significantly less active due to the smaller and more strongly binding internal cavity. Schmitzer and co-workers developed triphenylbenzimidazole metal–organic assemblies that function as chloride channels in the presence of Pd(ii).^78^ The authors reported an increase in chloride efflux in LUVs upon formation of the metal-templated assembly, and demonstrated that the assemblies could improve the permeability and efficacy of antibiotics to resistant bacteria without any significant toxicity.

#### Self-assembled ion and water channels

A common and frequently successful approach is the self-assembly of channels from individual monomers, which themselves are often simple and readily accessible compounds. The structures typically contain hydrophilic ion binding motifs, lipophilic components to maximise insertion into the bilayer, and recognition motifs such as hydrogen bonds to promote self-assembly. Channels assembled from monomers are arguably the most commonly employed strategy, with several examples reported for anions,^82^ cations^83^ and water,^84^ and more recently photoswitchable,^85^ caged^86^ and ligand gated^87^ variants. We highlight selected recent examples here.

**Fig. 9:**
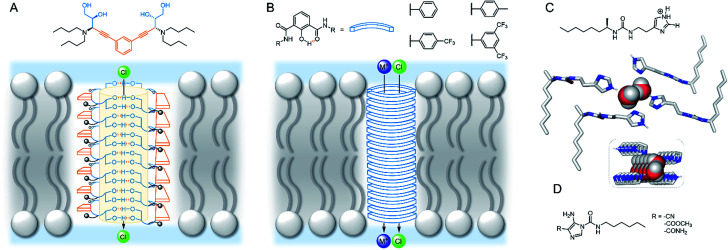
Self-assembled channels. (A) Bis-diol barrel-stave chloride channel^89^ (B) KCl/NaCl barrel-rosette channel;^90^ (C) imidazole ureas and solid state structure of an imidazole quartet water channel (CCDC: OMOTER);^91^ (D) pH regulated imidazole urea capable of water/chloride transport.^92^

Talukdar and co-workers utilised fumaramides that self-assemble *via* hydrogen bonds in a highly controlled fashion to form barrel-stave anion channels.^88^ Subsequent work included a series of 1,3-diethylenylbenzene spaced bis-diols, capable of self-assembling into tubular barrel-rosette-type anion channels *via* hydrogen bonds, with high chloride selectivity in vesicles (Fig. 9A).^89^ The authors observed a strong correlation between chloride transport in cells induced by the channel, and caspase-dependent apoptosis pathways, suggesting that the system may show promise in the context of cancer therapeutics. The same group also developed a barrel-rosette channel built from 2-hydroisophthalimides *via* H-bonds and π–π interactions, capable of facilitating KCl or NaCl symport (Fig. 9B).^90^ The authors successfully confirmed the roles of both cation and anion transport towards apoptotic pathways in cells incubated with the 3,5-bis-trifluoromethylaryl derivative, and were able to visualise the compound within live cells using the intrinsic fluorescent properties of the channel.

Hydrogen bonding self-assemblies have also been used to transport water across lipid bilayers. Barboiu and co-workers observed that imidazole linked ureas can stack and form pore sizes similar to Aqp aquaporin, leading to high water transport rates and salt rejection across lipid bilayers.^91^ Crystal structures revealed water wires surrounded by four imidazole stacks, with the chiral alkyl substituted derivative exhibiting improved water wire ordering and transport rates (Fig. 9C). The same authors demonstrated that single file alignment of water and chloride was possible when amino-imidazoles were directly attached to the urea (Fig. 9D), leading to high chloride conductance.^92^ The authors attribute the high ion transport activity to the possible acidic [H_2_O·Cl^−^] and basic [OH^−^·H_2_O·Cl^−^] binding to the water wires. Thus, an HCl symport mechanism dominates at low pH; OH^−^/Cl^−^ antiport at high pH; while at intermediate pH there is a minima in transport activity. The authors observed non-linear ohmic behaviour at low pH and especially high pH, and thus proposed that the antiport state creates a dynamic charge distribution across the channel externally stimulated by a strong electric field.

**Fig. 10:**
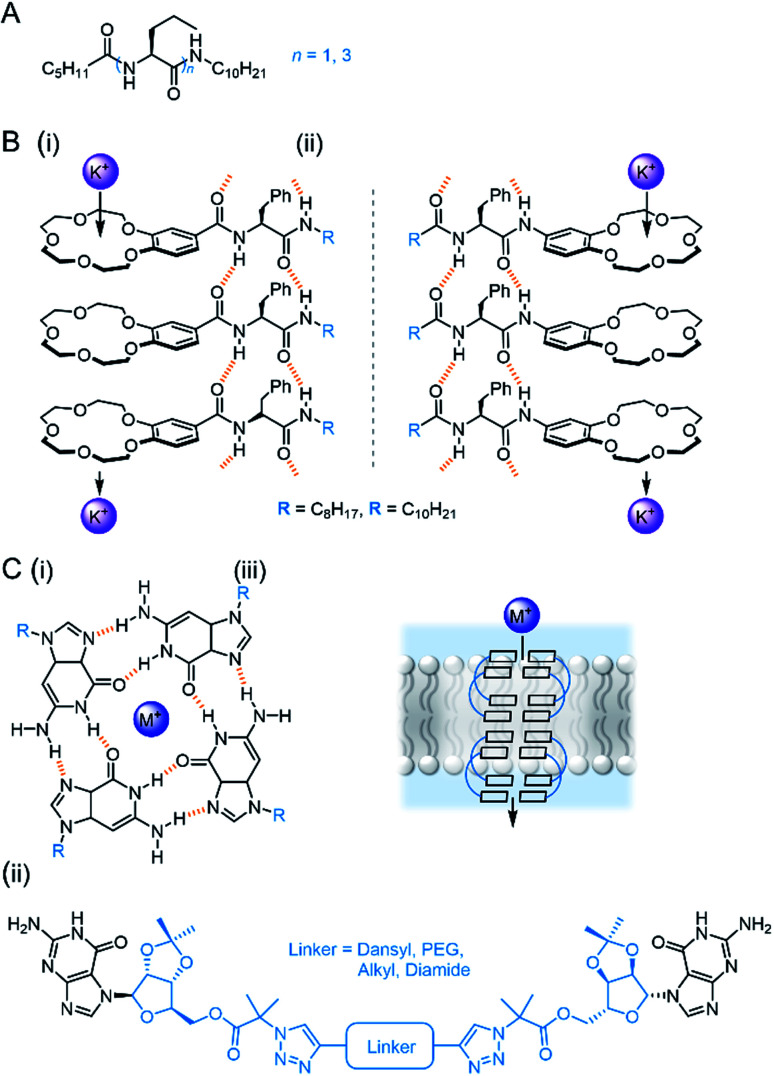
Bio-inspired self-assembled channels (A) Zeng's self-assembling peptides.^93,94^; (B) peptides modified with crown ethers (i = C-terminus modified, ii = N-terminus modified);^95^ (C) (i) Tetrameric guanosine quartet with a cation binding site; (ii) di-guanosine channel modified with various linkers; (iii) schematic of quartet forming within bilayer to form cation channel.^96^

Bio-derived self-assembling motifs have also successfully been employed within synthetic channels. Zeng and co-workers have demonstrated that mono- and tri-peptides with pendent alkyl chains form highly active anion channels *via* intermolecular H-bonds, with EC_50_ values as low as 0.005 mol% (channel with respect to lipid) in vesicles (Fig. 10A).^93,94^ The Li group exploited the reliable self-assembling properties of peptides to generate cation channels by appending benzo-crown ethers to the C- or N-terminus (Fig. 10B). The more electron rich N-terminus derivatives exhibited both improved activity and greater K^+^ > Na^+^ selectivity relative to the C-terminus derivatives, suggesting that cation binding is the more important determinant of activity compared to ion release.^95^

Guanosine derivatives are also known to self-assemble into a tetrameric G-quartet containing a cation binding site, which can stack *via* aromatic interactions to form a channel (Fig. 10C).^96^ Inspired by this, Dash and co-workers developed linked bis-guanosine derivatives which reliably formed helices to mediate Na^+^, K^+^ and Cs^+^ transport (Fig. 10C(ii)). Linkers included a dansyl unit for imaging within the bilayer, diamides for improved self-assembly, a lipophilic derivative for enhanced membrane deliverability and PEG linker to form G-quadruplex amphiphilic ion channels.^96^

#### Unimolecular ion channels

A channel formed from a membrane-spanning single molecule is arguably more similar to naturally occurring channel proteins, but are structurally simpler and less likely to suffer from issues such as mis-folding, denaturing or difficulty in isolating large quantities. Unimolecular channels may also require lower doses compared to self-assemblies made up from monomers, which are concentration dependent.^97^ Moreover, a unimolecular channel will stay intact, as opposed to a reversibly formed self-assembled channel. Membrane-spanning single molecule channels have been successfully employed in both synthetic and natural membranes, using either artificial supramolecular architectures or biological building blocks such as peptides.^98^

**Fig. 11:**
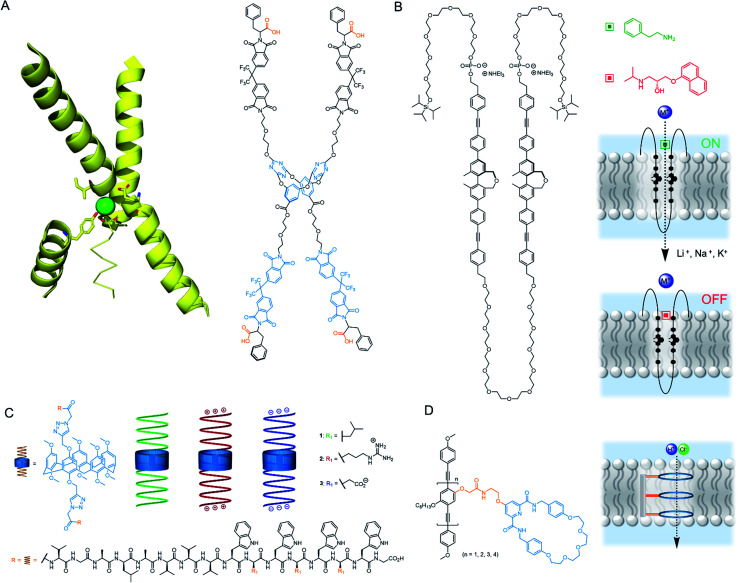
Unimolecular channels (A) CLC chloride channel with key helical domains (PDB: 1KPL) and CLC mimic;^99^ (B) Multiblock amphiphile by Kinbara with anisotropic ligand response^100^ (C) Gramicidin-modified pillararenes by Chen^103^ (D) Rigid rod ion channels reported by Zhu and co-workers.^104^

In 2020, Wang and co-workers developed an artificial chloride selective channel inspired by the hourglass shape of the natural CLC channel (Fig. 11A).^99^ The electron deficient triazine-based macrocycle appended to four electron deficient imides leads to high anion transport activity *via* anion–π interactions, and organises the system into the “hour glass” conformation. The high Cl^−^ > Br^−^ selectivity was attributed to the narrow macrocyclic cavity preferring the smaller and more strongly bound Cl^−^, leading to exclusion of incoming Br^−^. The carboxylate group was key for activity; which was proposed to improve water solubility, anchoring to the polar lipid head groups, and also help maintain the “hour glass” conformation due to inter-anion repulsion.

The research group of Kinbara have developed a variety of single molecule ion channels that respond to external stimuli such as ligands,^100^ electric fields^101^ or mechanical stress.^102^ An impressive example includes a multi-block amphiphile (Fig. 11B), which inserts itself into the bilayer in a controlled orientation due to its amphiphilic nature; allowing gating of ion transport activity with both agonistic and antagonistic ligands.^100^ The charged phosphate groups function as a binding site for aromatic amines and ensure high dispersion in aqueous environments. The structure was found to adopt a folded conformation in water and lipid bilayers, with the chiral biphenyl units in close proximity. When added externally to pre-formed membranes, both phosphate groups resided within the extra-vesicular medium, whereas pre-incorporation resulted in an alternative inactive conformation. The amphiphile behaved as a cation channel (Li^+^ > Na^+^ > K^+^) in the presence of 2-phenylethylamine, whereas propranolol could block the hydrophobic pore and switch off ion transport. Although the full molecule spans the lipid bilayer, Hill analysis suggests it functions when three molecules assemble in the bilayer. In living cells, the authors provided evidence for incorporation of the structure within the bilayer and were able to successfully activate Ca^2+^ transport in the presence of 2-phenylethylamine.

Chen reported a series of pillararene derivatives modified with gramicidin, a helical peptide capable of efficiently transporting K^+^ (Fig. 11C).^103^ The negatively charged C-termini derivative displayed the highest activity in planar bilayer experiments due to the attractive charges towards K^+^. The neutral analogue performed best within LUV assays, whereas the positively charged derivative possessed the highest antimicrobial activity (IC_50_ = 0.55 μM), which is possibly a consequence of the strong interaction with the negatively charged bacterial membrane. The compounds displayed minimal haemolytic toxicity, thus showing promise as potential antibiotics. Finally, Zhu and co-workers reported a series of anion-binding pyridine carboxamide macrocycles attached to rigid rods of varying length (Fig. 11D). When *n* = 3, the rod length was complementary to the thickness of the lipid bilayer, resulting in efficient HCl symport through the unimolecular channel.^104^

#### Aromatic foldamers

**Fig. 12:**
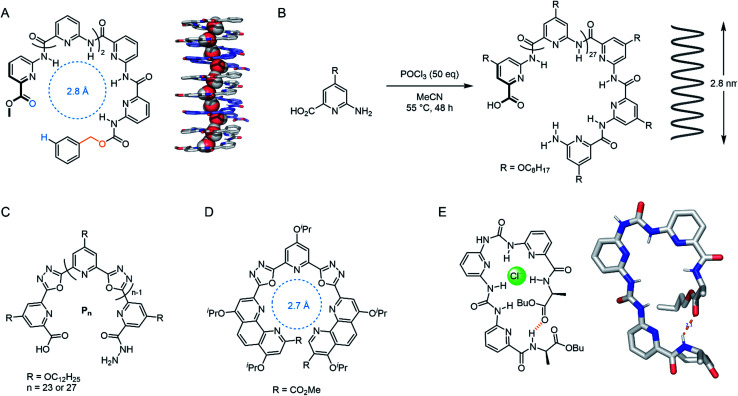
Foldamer-based channels. (A) ‘Sticky-end’ modified polypyridine for water transport, with solid state structure of the 5-mer assembly, co-crystallised with MeOH (CCDC: BUHZEM).^106^ (B) Unimolecular polypyridine foldamer capable of spanning the length of lipid bilayer.^107^ (C) Unimolecular cation channel: *n* = 27 derivative spans the bilayer.^108^ (D) Highly active and K^+^ selective isoxazole foldamer monomer.^109^ (E) Pseudo-macrocycle foldamer, capable of stacking into a Cl^−^ channel, with solid state structure (CCDC: IQENUQ)110.

Foldamers are designed to adopt a predictable and compact conformation using non-covalent interactions, typically derived from non-biological building blocks.^105^ Aromatic foldamers are attractive motifs for both synthetic cation, anion and water channels as they reliably form helical structures in aqueous solution. This is generally accomplished *via* π–π interactions or hydrogen bonding. For example, Zeng and co-workers reported a poly-pyridine derivative that functions as a highly active water channel with high ion rejection (Fig. 12A).^106^ The alternating ‘sticky ends’ facilitated the controlled self-assembly of a highly stable helix containing alternating hydrogen bond donors and acceptors within a 2.8 Å pore; complementary to the spatial and electronic requirements of water and matching the size of natural AqpZ. The low ion permeability was attributed to the tight pore size and lack of ion binding motifs. A unimolecular derivative that spans the full bilayer using a one-pot polymerisation (Fig. 12B) was also reported.^107^ The foldamer was able to transport H_2_O and H^+^ with extremely high selectivity, with proton transport rates approaching that of the natural proton channel gramicidin.

The same group have also reported a membrane-spanning unimolecular oxadiazole foldamer, which behaves as a cation channel with 16-fold K^+^ > Na^+^ selectivity (Fig. 12C).^108^ The longer, fully membrane-spanning polymer (*n* = 27) displayed higher transport activity but poorer selectivity relative to the shorter derivative (*n* = 23). This year, Dong and co-workers reported a related derivative in which the outer pyridines were systematically replaced with phenanthrolines, tuning the pore size to 2.7 Å (Fig. 12D). This provided up to 30-fold K^+^ > Na^+^ selectivity and very high transport activity.^109^ Wu and co-workers designed *meta*-pyridine-urea derivatives that form a pseudo-macrocycle/foldamer (Fig. 12E). These compounds were found to reliably stack inside the lipid bilayer *via* hydrogen bonding, forming a chloride-selective channel (0.5 mol% transporter relative to lipid).^110^ Very recently, Zeng and co-workers have also optimised their water transport foldamer systems further to achieve remarkable H_2_O transport rates outperforming aquaporins, with proton exclusion, by using a membrane-spanning foldamer with a hydrophobic pore.^111^

### Mobile ionophores

**Fig. 13:**
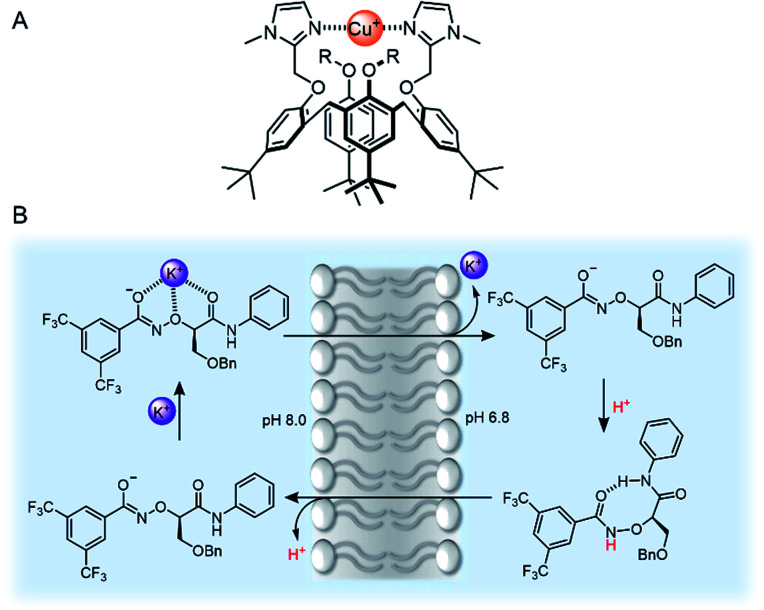
(A) Bis-imidazole-functionalised calix[4]arene Cu(i) transporter.^115^ (B) Mechanism of action of Yang's potassium transporter.^116^

As we have discussed, there are many examples of synthetic channels which facilitate cation transport, but somewhat surprisingly, mobile cation carriers are rarer. Well-known examples include the bacterial natural products valinomycin and calcimycin. Nevertheless, examples of synthetic carriers for a range of group I, II and transition metal cations including Zn(ii)^112^ and Cu(ii)^113,114^ are known and are of interest as anti-cancer agents. A recent advancement in copper transport was reported by Valkenier and co-workers, who demonstrated the first example of a mobile carrier for Cu(i) cations mediated by bis-imidazole-functionalised calix[4]arenes (Fig. 13A).^115^ To quantify Cu(i) transport, the authors developed a new assay in LUVs in which the fluorescent dye bathocuproine disulfonate (BCS) was encapsulated within the vesicles. Transport of Cu(i) across the vesicle membrane (added as Cu(ii) in the presence of a reducing agent) is reported by fluorescence quenching of BCS, due to complexation of Cu(i) in a 2 : 1 receptor:cation stoichiometry.

Recent advances in group I cation transport have also emerged, driven by the observation that potassium transport in particular is often impeded as a result of channelopathies, and implicated in certain cancers. Yang and co-workers reported an elegant example of mobile carriers for potassium,^116^ which incorporated an α-aminoxy acid motif that they had previously employed within a potassium selective synthetic ion channel.^117^ The carrier selectively transports K^+^ (*via* a K^+^/H^+^ antiport mechanism, Fig. 13B) within the typical *in vitro* transport assays, but also across lysosomal and mitochondrial membranes in living cells. This leads to enhanced cytotoxicity to chemo-resistant ovarian cancer stem cells (CSCs) and supressed tumour formation in mice. The selectivity for potassium is attributed to the electron-withdrawing effect of the bis-CF_3_ substituents, which increases the acidity of the aminoxy amide NH proton.

### Amino acid transporters

**Fig. 14:**
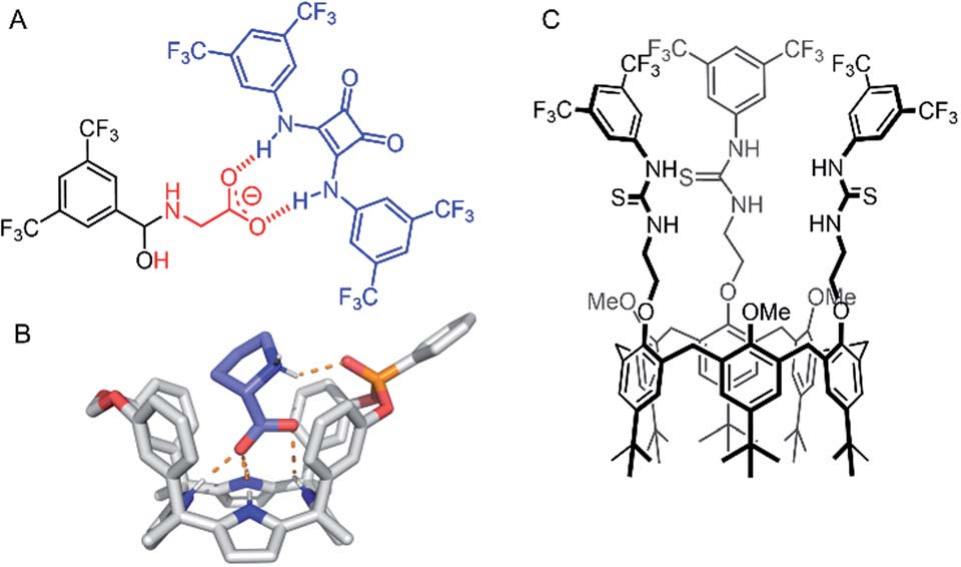
Amino acid and ion pair transporters. (A) Glycine transporter.^119^ (B) Crystal structure of calix(4)pyrrole cavitand transporter with bound l-proline (CCDC: XAHRUX).^120^ (C) Calix[6]arene tris(thio)urea ion-pair transporter.^121^

Amino acid transport across lipid bilayers requires the assistance of a carrier protein (known as amino acid transporters, AATs) in the process of facilitated diffusion. Synthetic examples remain extremely rare, but in recent years, a number of approaches have emerged. Hou and co-workers have reported peptide-appended pillar[*n*]arene (*n* = 5, 6) derivatives that act as transmembrane channels for the enantioselective transmembrane transport of amino acids.^118^ More recently, Gale and co-workers have presented a novel approach to amino acid transport that exploits dynamic covalent and non-covalent interactions to mediate transport.^119^ Glycine transport was facilitated by hemiaminal formation of the amino acid amine with a simple electron deficient aldehyde embedded in the membrane, and hydrogen bonding of the carboxylate motifs to a squaramide anionophore (Fig. 14A). In this study, amino acid transport was examined using a ^13^C NMR assay with ^13^C- enriched glycine, as well as a fluorescence assay in which the transported amino acid displaces Cu(ii) bound to calcein, restoring fluorescence. Control experiments show the necessity of both the squaramide and the aldehyde for effective glycine transport. The most recent example of an amino acid transporter was reported by Ballester, Palacin and co-workers, in which the facilitated diffusion of l-proline (l-Pro) across a membrane is mediated by a calix(4)pyrrole cavitand (Fig. 14B).^120^ The cavitand was designed such that the arrangement of the NH hydrogen bond donors (for carboxylate binding) and the phosphine oxide hydrogen bond acceptor are complementary for l-Pro binding. Transmembrane transport of l-Pro was determined using a ^13^C NMR assay, and unusually, with [^3^H] radiolabelled amino acids, which were employed in radiometric influx and efflux experiments. The transport assay was stopped at specific intervals by adhering the vesicles to nitrocellulose filters, and the radioactivity arising from trapped labelled amino acids quantified with a β-counter.

### Ion-pair transporters

Ion-pair carriers are attractive for applications involving the selective transport of ions, such as the coupling of transport of an anion to an existing cellular cation gradient. However, examples remain rare,^122–124^ in part due to the difficulties in the selective binding of both a cation and an anion within the same receptor, and also due to the inherent challenges of determining the transport mechanism in a multi-ion transport process. The first example of an ion pair transporter was reported in 2003 by Smith and co-workers, in which sodium or potassium chloride transport as a contact ion-pair was facilitated using a macrobicycle with a diaza-18-crown-6 motif for cation binding and an isophthalamide hydrogen bond donor for simultaneous chloride binding.^122^ More recently, Valkenier and co-workers reported a calix[6]arene tris-thiourea derivative capable of transporting organic ion pairs.^121^ The transporter features a calix[6]arene cavity for propylammonium binding, appended with three pendant urea or thiourea motifs for anion binding (Fig. 14C). Transport assays were conducted using variations of the lucigenin assay, a fluorescent dye that is quenched by chloride. These experiments demonstrated that transport of chloride only occurs in the presence of a propylammonium cation *via* transport of the ion pair.

### Oxo-anion transporters

**Fig. 15:**
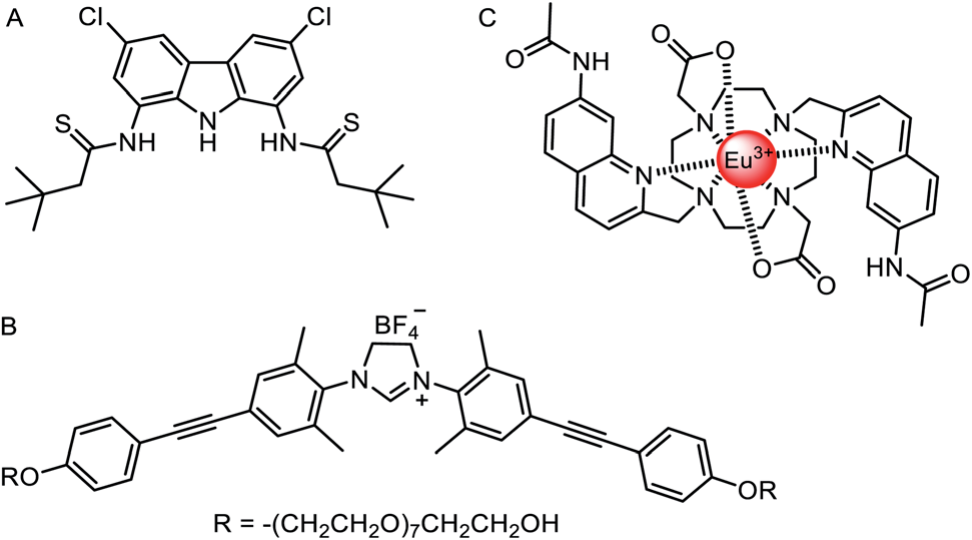
Oxo-anion transporters. (A) Thioamide carbazole-based oxo-anion carrier.^126^ (B) Nitrate-selective imidazolium-based mobile carrier.^128^ (C) A europium(iii) complex for direct measurement of bicarbonate transport in LUVs.^129^

The vast majority of synthetic anionophores reported to date facilitate chloride transport. Surprisingly, examples of anion carriers capable of mediating oxo-anion transporters are comparatively rare, perhaps in part due to a historic lack of suitable assays, or the complexity of studying transport of species that can exist in multiple protonation states. Chmielewski and co-workers have reported a series of bis-amide derivatives of 1,8-diaminocarbazoles and 3,6-dichloro-1,8-diaminocarbazoles.^125^ Their readily accessible structures proved to be tuneable ‘turn-on’ fluorescence sensors for H_2_PO_4_^−^ and AcO^−^, and potent transmembrane transporters of chloride. The thioamide carbazole derivative (Fig. 15A) was shown to be effective for the transport of various biologically relevant oxyanions such as aspirin and pyruvate, as shown *via* the versatile lucigenin assay in both LUVs and GUVs.^126^

As discussed above, Kinbara and co-workers have pioneered the use of multi-block amphiphiles as ion channels.^127^ However, they recently have also reported a related imidazolium derivative, which acts as a mobile anion carrier (Fig. 15B).^128^ The structure consists of an imidazolinium anion binding site, positioned between diphenylacetylene units appended with non-ionic, hydrophilic octa(ethylene glycol) chains. This anionophore mediated nitrate selective transport with a selectivity trend of NO_3_^−^ > ClO_4_^−^ > Cl^−^ > Br^−^ > I^−^.

Ion transport assays in lipid bilayer vesicles are an indirect measurement of the overall process, typically quantifying changes in intra- or extra-vesicular ion concentrations with time, using the fluorescence response of ion-responsive fluorophores or an external ion selective electrode. Whilst such assays are convenient and widely used, care must be taken in the interpretation of the results, in particular when a number of different mechanisms may be taking place. Bicarbonate transport data has proved especially challenging to interpret. However, Valkenier, Butler and co-workers^129^ have reported a new assay that enables the direct measurement of bicarbonate transport, which previously could only be inferred in coupled transport assays (*e.g.* monitoring chloride transport rates during chloride/bicarbonate exchange).^130^ Their assay employed a water-soluble europium complex (Fig. 15C) which exhibits a large increase in emission intensity upon bicarbonate binding at physiologically relevant millimolar concentrations. Crucially, the europium complex has high selectivity for bicarbonate anions over chloride and nitrate, which are typically used in anion transport assays. Using a previously reported bambusuril macrocycle as a test anionophore, the authors were able to use the assay to directly measure the kinetics of bicarbonate transport,^131^ and distinguish between other mechanisms involving CO_2_ diffusion and pH gradient dissipation (which also led to an increase in bicarbonate concentration inside vesicles without transport of the anion).

### Anionophores employing sigma hole interactions

**Fig. 16:**
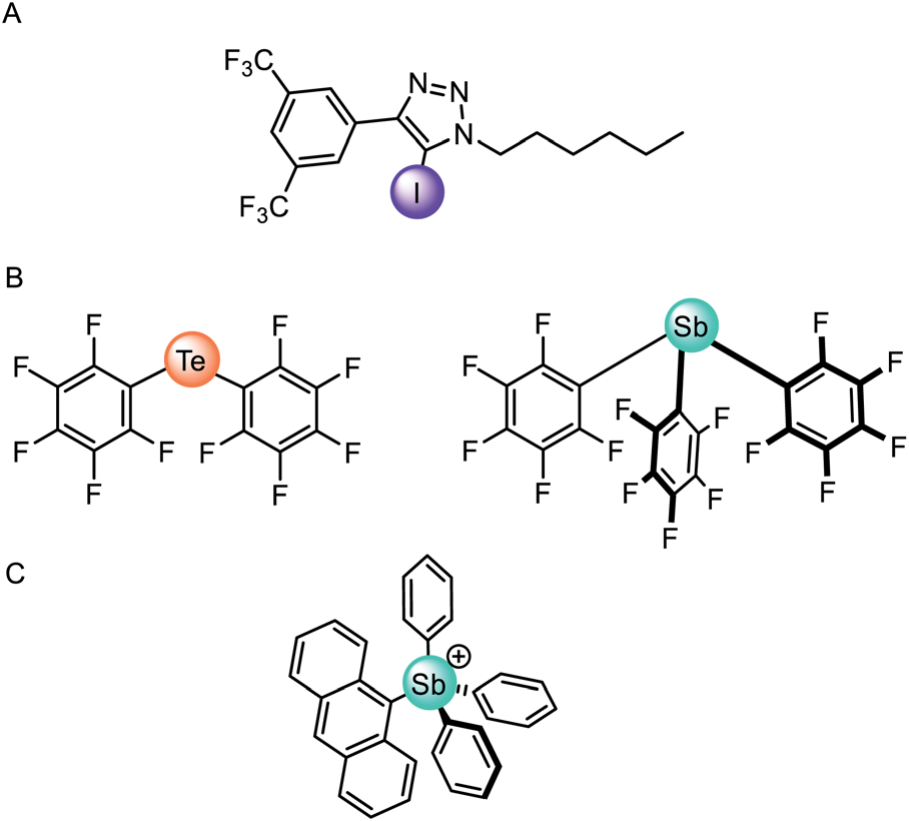
(A) Monodentate iodotriazole halogen bonding anionophore.^138^ (B) Neutral chalcogen and pnictogen anion carriers.^139^ (C) Pnictogenium cation anionophore.^140^

The vast majority of synthetic anionophores employ polar acidic hydrogen bonding motifs, typically NH donors, for anion recognition and transport. However, the use of non-classical intermolecular interactions for mediating anion transport, including CH hydrogen bonding,^132^ anion–π,^133^ and sigma hole^134^ interactions, have emerged as effective alternatives. Sigma holes are electron-deficient regions which arise from anisotropic distribution of electron density on a main-group atom bonded to an electron withdrawing group, and leads to the formation of halogen (group 17), chalcogen (group 16) and pnictogen (group 15) bonding interactions, respectively.^135^ These interactions are typically less hydrophilic than polar NH or OH hydrogen bond donors, and thus experience diminished dehydration penalties for transport and are less inclined to promote detrimental transporter aggregation. The use of these exotic non-covalent interactions for membrane transport was pioneered by Matile and co-workers,^136^ who demonstrated, for example, that simple halogen bond donors such as iodo-perfluorobenzene and iodo-perfluorohexane are effective anion transporters.^137^ Langton and co-workers later introduced iodotriazoles as chloride selective halogen bonding anionophores with activity surpassing both the previous halogen bonding systems and the analogous CH hydrogen bonding prototriazole anionophores (Fig. 16A).^138^ Computational studies revealed the unique ability of the halogen bonding anionophores to delocalise the anion charge over the complex, which correlates with the enhanced anion transport capability observed experimentally.

Recently chalcogen (group 16) and pnictogen (group 15) bonding interactions have also found application within the field of anion transport. Matile and co-workers compared a family of electron deficient chalcogen and pnictogen bonding anionophores (Fig. 16B), in which the tellurium-centred chalcogen bonds were more active than the halogen bonding analogue.^139^ This was attributed to the presence of two sigma-hole donors (as opposed to just one found in halogen bonding derivatives), and the proximity of the π-acidic perfluorobenzene surfaces for secondary anion–π interactions. In contrast, strong anion binding to the tris(perfluorophenyl)stibane pnictogen bonding receptor led to the formation of membrane-disruptive supramolecular amphiphiles.

Gabbaï and co-workers have also reported a variety of main-group anionophores, including pnictogenium cations^140^ and phosphonium boranes.^141^ For example, they reported the ability of tetraaryl-stibonium and tetraaryl-bismuthonium cations (general formula [Ph_3_PnAr]^+^, where Pn = Sb or Bi and Ar = phenyl, naphthyl, anthryl, or pyrenyl) to transport fluoride anions in LUVs and human erythrocytes cells (Fig. 16C).^140^ The same group also demonstrated that the transport activity of diaryltellurides Mes(C_6_F_5_)Te and (C_6_F_5_)_2_Te is enhanced following oxidative methylation to form [Mes(C_6_F_5_)TeMe]^+^ and [(C_6_F_5_)_2_TeMe]^+^, respectively.^142^ This is rationalised by the lowering of the tellurium-centred σ* orbitals and deepening of the associated sigma-holes, leading to increased Lewis acidity of the chalcogen bond donor. These observations demonstrate that controlling the redox states of main group elements provides a handle to modify the properties of chalcogen bond donors.

### Achieving anion selectivity

**Fig. 17:**
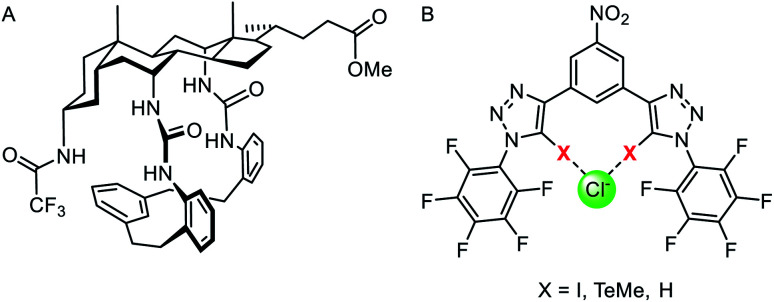
(A) Macrocyclic cholaphane anion carrier.^143^ (B) Halogen (X = I), chalcogen (X = TeMe) and hydrogen bonding (X = H) bidentate anionophores.^144^

Two main strategies have been explored to achieve anion selectivity within mobile anion carriers: geometric complementarity and tuning of the Lewis acid site (hydrogen bond donor acidity or employing Lewis acidic sigma hole interactions). Geometric complementarity between the transporter and anion is desirable for selective ionophores, but is more often observed in synthetic ion channels in which the pore size is tuned appropriately for the ion of choice. However, Davis and co-workers reported a macrocyclic derivative of their cholaphane mobile carriers exhibiting enhanced transport activity and selectivity (Fig. 17A).^143^ The cyclic cholaphanes – capable of encapsulating the anion in a complementary binding pocket – displayed enhanced selectivity for chloride over nitrate by a factor of up to 2.5 relative to their acyclic cholapod analogues. Moreover, the cyclic ionophores were more active than their acyclic counterparts within a chloride/nitrate exchange assay. Justification for these findings is two-fold: selective encapsulation of the target anion to exclude other potential anionic guests, and secondly, improved shielding of the anion charge from the hydrophobic membrane, which leads to faster transport kinetics. A later study on the same system, in comparison to a wide range of known anionophores, demonstrated that the macrocyclic cholaphane exhibited very high (∼100 fold) chloride over proton/hydroxide transport selectivity.^145^ Such selectivity is likely to be critical for applications such as treatment of channelopathies, as unintended dissipation of cellular pH gradients by anionophores may lead to toxicity. The authors demonstrated that a combination of chloride anion encapsulation, and the absence of highly acidic hydrogen bond donors, is necessary to achieve such selectivity.

Langton and co-workers have subsequently shown that the selectivity of an anionophore may be tuned by varying the nature of the intermolecular interaction at work. They reported structurally similar bidentate halogen and chalcogen bonding carriers derived from iodotriazole and telluoromethyl triazoles respectively (Fig. 17B).^144^ Anion transport experiments in vesicles revealed that bidentate halogen bonding systems were very active, up to three orders of magnitude greater than previously reported sigma hole anionophores.^138^ The chalcogen bonding anionophore was also highly active and demonstrated high (∼70-fold) chloride over hydroxide transport selectivity, without requiring a macrocyclic structure or weakly acidic hydrogen bond donors (which may lead to poor activity due to weaker anion binding).

### Stimuli-responsive transport systems

In nature, transmembrane ion channels and pumps are responsive to chemical or physical changes in their environment. For example, the generation of an action potential in a neuron is triggered by the binding of small-molecule ligands to responsive ion channels or changes in membrane potential,^146^ while rhodopsin ion channels are stimulated by light.^147^ Whilst the fundamentals of abiotic passive ion transporters have been widely explored, significantly less attention has been focused on controlling synthetic transporters. This emerging field has been recently reviewed by one of us,^148^ and so we highlight only a handful of representative examples of responsive mobile channels and carriers here.

**Fig. 18:**
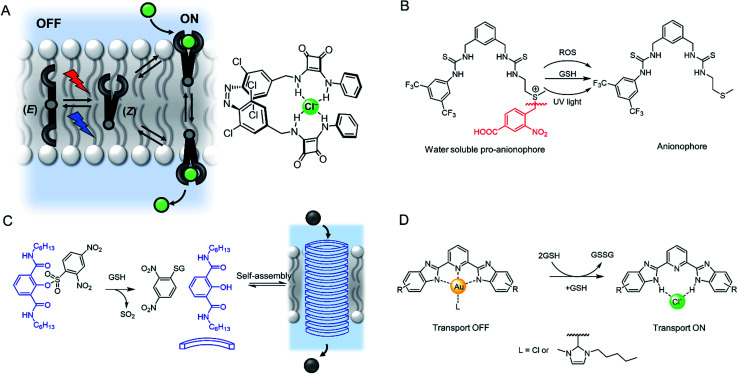
Stimuli-responsive ion transporters. (A) Visible-light switchable ion carrier for reversible control over ion transport.^149^ (B) A sulfonium-linked procarrier activated irreversibly by reactive oxygen species (ROS), glutathione (GSH) or UV light.^150^ (C) A GSH-activatable ion channel.^151^ (D) Responsive gold complexes.^152^

Light is an attractive stimulus for controlling ion transporters and channels, due to the possibility of adding and removing the stimuli with spatio-temporal control, and good bio-orthogonality. Recently a number of photo-responsive ionophores have emerged utilising azobenzene photo-switches. Kerckhoffs and Langton reported red-shifted azobenzenes appended with squaramide anion binding motifs, which could be switched reversibly between the transport-inactive *E* isomer and transport-active *Z* isomer using biocompatible red light (Fig. 18A).^149^ This is in contrast to previously reported azobenzene based ionophores which are triggered by UV light, which suffers from poor biocompatibility.^153^ By irradiating a sample of LUVs containing the photo-switchable transporter, and simultaneously measuring chloride transport using an ion selective electrode, the authors demonstrated that the chloride transport activity could be reversibly turned on and off by *in situ* photo-isomerisation of the carrier in the membrane.

An alternative method is to “cage” the anionophore. This can be achieved by either blocking the binding site to prevent anion recognition and transport until the cage is removed by the stimulus, such as with a photo-labile protecting group,^154^ or by appending a hydrophilic cage (*e.g.* a polar sugar) that prevents the carrier partitioning into the lipid bilayer until removed by a stimulus.^155^ An example of the latter approach has been reported by Manna and co-workers, who showed that a water soluble and inactive sulfonium-based pre-anionophore could be made membrane permeable by reduction with glutathione, leading to activation of anion transport (Fig. 18B).^150^ Chloride transport could also be stimulated by reactive oxygen species or by irradiation with 365 nm light. Cancer cells over produce ROS and GSH thus this chloride specific carrier may be suited to applications within targeted cancer therapy. In a related approach, Gabbaï and co-workers reported a dicationic hydrophilic stibonium/sulfonium pro-carrier, which is also reduced by glutathione to form a mono-cationic, lipophilic pnictogen bonding chloride carrier.^156^

A similar strategy employed to control ion channels has been reported by Talukdar and co-workers, who prepared a 2,4-dinitrobenzenesulfonyl (DNS) protected 2-hydroxy isophthalamide (Fig. 18C).^151^ In the presence of glutathione, the hydroxyl group is exposed, leading to self-assembly of a M^+^/Cl^−^ channel, which was capable of inducing cell death in MCF-7 cells. The same group also reported a related analogue activated through cleavage by esterases, which are overexpressed in cancer cells, and observed both apoptosis activation and disruption of autophagy.^157^

Gale and co-workers have reported an alternative “caging” approach, in which a metal cation blocks the anion binding site of an anionophore until it is displaced (Fig. 18D). Their system involved a bis-imidazole-based anion transporter coordinated to Au(iii), unable to perform anion transport until activated by decomplexation of the gold in the presence of a reducing agent such as glutathione (GSH), allowing an anion to bind and membrane transport to occur.^152^ This novel mechanism for switchable transporters was used in cell viability studies that showed the caged carriers to be less cytotoxic than their ‘switched-on’ analogues, highlighting the potential for this work to be adapted for biomedical applications.

The presence or absence of metal ions provides a means to gate the activity of metal-templated self-assembled ion channels. For example, Zhu, Liu and co-workers designed an artificial phenanthroline derivative, which formed a hollow helical cation channel *via* intermolecular π–π interactions and intramolecular H-bonds.^158^ In the presence of Cu(i), the phenanthrolines rearranged into a double helix lacking an internal cavity; thus switching off ion transport. Upon addition of ammonia to remove the Cu(i), it was possible to restore the original channel activity.

**Fig. 19:**
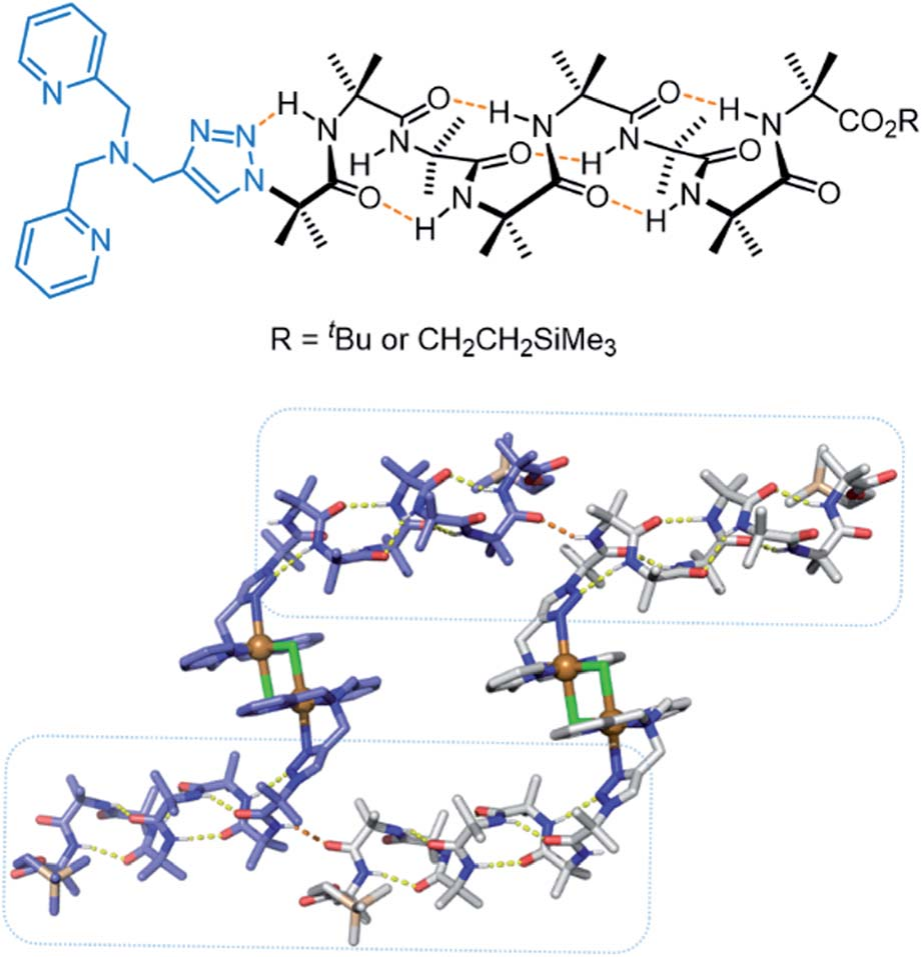
Aminoisobutric acid helix and dimeric crystal structure (CCDC: PURQOL).^159^ Extended crystal structure also shown for another Cu(ii)-helix unit (blue atoms).

More recently, Clayden, Cockroft, Webb and co-workers developed an aminoisobutric acid helix which forms a dimeric structure in the presence of Cu(ii) (Fig. 19).^159^ The extended crystal lattice revealed that the dimers form head-to-tail hydrogen bonds, thus forming the basis of a chloride channel. In LUV assays, the authors demonstrated switching ‘ON’ of transport when CuCl_2_ was added, whereas the activity was switched ‘OFF’ by removing the Cu(ii) with EDTA. The Cu(ii) complex of the helix displayed potent antibacterial properties with minimal haemolytic activity, which the authors attribute to the lower hydrophobicity relative to other antibiotics.

## Future prospects

As we have seen, the field of supramolecular chemistry in membranes has advanced significantly in recent years. Many of the cornerstones of supramolecular chemistry in solution are finding new applications in biological contexts by interfacing with membranes; including molecular recognition, self-assembly, supramolecular catalysis, foldamers, molecular switches and molecular machines. Here we provide a personal and somewhat speculative viewpoint on where we think the future challenges and opportunities lie in the short to medium term. We envisage three areas in which these developments will come: (1) the fundamentals: new molecular designs, functions, out-of-equilibrium behaviour, understanding and experimental techniques; (2) applications in complex artificial systems, such as synthetic cells, multi-component networks and soft materials; and (3) applications in biology and medicine.

### Understanding molecular self-assembly in membranes

Molecular recognition and self-assembly within bilayers is the basis for all the applications we have explored in this perspective. However, in comparison to solution phase supramolecular chemistry, a detailed understanding of these processes in membranes is surprisingly lacking. As discussed earlier, the effective concentration of supramolecular systems embedded in a bilayer are often many orders of magnitude higher than in bulk solution because they are confined within effectively a two dimensional membrane. Understanding, and – just as importantly, if not more so – predicting self-assembly and recognition processes within the bilayer is crucial. A major challenge here is a lack of convenient experimental techniques to probe self-assembly. For example, the mode of action of a self-assembled ion channel is inferred from the observation of ion flux, and little structural information from transport assays can be obtained. Techniques used extensively in solution phase supramolecular chemistry such as 2D NMR methods are unavailable, and the transport-active species may not be thermodynamically stable. Interpretation of the frequently used Hill coefficients obtained from the Hill equation during analysis of dose–response curves is often difficult, because they can be highly sensitive to minor structural changes, and rely on the transport system to be thermodynamically unstable in order to determine stoichiometry.^160^ We envisage increasing use of advanced *in silico* experiments, such as molecular dynamics simulations within model membranes, to assist in the design of new structures, interpret experimental data and probe structural properties. Whilst computational modelling of membrane-bound proteins is a well-established field, modelling synthetic supramolecular systems in membranes is in its infancy and typically confined to studies on mobile ion carriers, such as those reported by Felix and co-workers.^161^

### Experimental techniques and assay

In recent years we have seen the emergence of many new assays which have shed light on the mechanism of action of existing systems^129^ or enabled the development of transporters for under-explored cargo.^119^ Fluorescence assays, ion selective electrodes and black lipid membrane conductance experiments are the primary tools, and if carefully interpreted^162^ with a plethora of suitable control experiments, provide extensive information on mechanism. The recent introduction of NMR-based transport assays and the use of radio-labelled cargo^120^ has also added to the toolbox of techniques. However, assays for the transport of more complex biologically relevant molecules such as peptides are also required. In this area, Hennig, Nau and co-workers have recently introduced dye-displacement assays in which transport of a cell-penetrating peptide or other biomolecule leads to displacement of a dye from a macrocyclic host such as cucurbituril (CB), concomitant with an observable fluorescence change.^163^ A related system has also been reported in which formation of a ternary complex of the analyte, CB8 and a reporter dye lead to a fluorescent response.^164^ Paegel and co-workers have very recently introduced an assay for quantifying the (non-facilitated) diffusion of alkyne-labelled amino acids in continuously generated microfluidic droplet interface bilayers.^165^ Copper catalysed azide–alkyne click reaction of the amino acids with encapsulated azides leads to a fluorescent response, and interestingly revealed their enantioselective permeation through a chiral phospholipid bilayer. Further progress in assay development will no doubt be driven by the desire to explore not only the facilitated transport of simple inorganic ions, but also more complex small molecules, biomolecules and therapeutics in the future.

### New designs and mechanisms for transport and signal transduction

We anticipate that many more novel systems for controlling ion transport, signal transduction and catalysis will emerge. We have already seen new abiotic mechanisms for signal transduction and amplification,^58^ and these and other new systems will no doubt advance into more sophisticated signalling systems able to more closely rival their biological counter-parts. The theme of controlling membrane-confined supramolecular systems also encompasses synthetic ion transporters as discussed above.^148^ Here the challenge is achieving spatial-temporal control, with well-defined inactive (OFF) and active (ON) states, using a range of stimuli that are appropriate for the desired application. In particular, targeted ionophores that can be activated in a tumour – by light, redox environment or membrane potential, for example – or targeted to a particular region of the body, will be key longer-term aims.

**Fig. 20:**
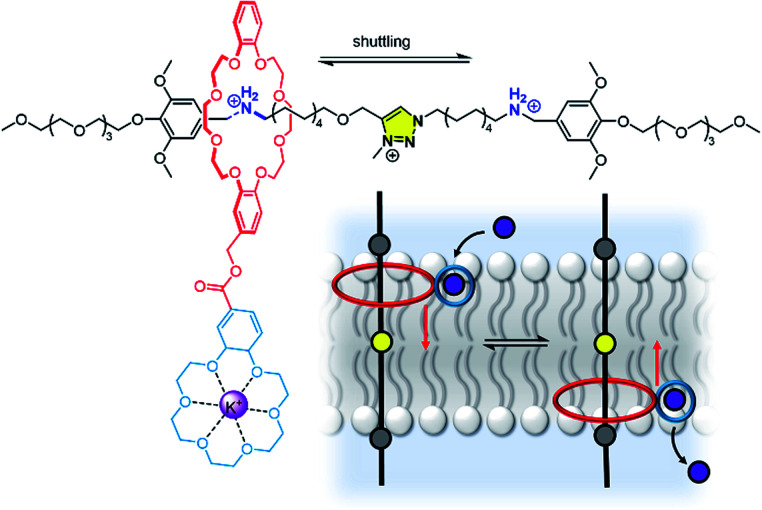
A transmembrane rotaxane ion transporter.^167^

The distinction between a mobile carrier and a membrane-spanning channel has blurred in recent years with the development of novel mechanisms for ion transporters, in particular molecular machines.^166^ For example, Bao, Qu, Zhu and co-workers have reported a membrane-spanning molecular shuttle capable of transporting potassium across a lipid bilayer (Fig. 20).^167^ The axle of the rotaxane features two ammonium salts as stations, with a triazolium intermediate station. The 24-crown-8 macrocycle capable of shuttling across the axle is appended to an 18-crown-6 unit for K^+^ binding and transport. This design has since been adapted very recently for photo-regulation by incorporating an azobenzene photo-switch into the axle.^168^ Photo-isomerisation from *trans* to *cis* inhibits shuttling and impedes the ion transport. These systems cannot be classified as a channel or mobile carrier, and instead combine aspects of both. Tour and co-workers have also used a molecular machine, in this case a photo-driven molecular rotor, to facilitate transport by “drilling” transient pores within the membrane.^169,170^ Experiments in vesicles and cells revealed that the membrane permeabilization, as well as cellular internalization of the motors, is enhanced when unidirectional rotation was induced by photo-irradiation. These molecular machines in membranes may serve as inspiration for gated channels or even systems capable of active transport, especially if further molecular machine designs capable of responding to external stimuli are developed.^171^

Out-of-equilibrium chemical systems have begun to emerge in recent years, such as dissipative self-assembly and chemically fuelled molecular machines.^172,173^ However, examples of membrane-bound systems that also function out-of-equilibrium are extremely rare,^148,174^ with fully synthetic examples limited to a handful of photo-driven ion transport systems discussed earlier,^43,44^ in addition to very recent developments by Gale in which a chemical fuel was used to generate proton gradients for driving ion transport.^175^ Significant focus in this area is now required to overcome the substantial challenge of developing fuel-driven membrane bound molecular machines, such as those capable of active transport across bilayers.

### Applications in complex artificial systems

The vast majority of artificial supramolecular systems that function in membranes are studied in relatively simple vesicles or planar lipid bilayers. However, if we look to the fields of synthetic biology, drug delivery and soft materials for inspiration, we find many different examples of artificial membrane-confined systems. Many of these may be suitable for interfacing with supramolecular systems to devise new functions, build complexity and target new applications.

The field of membrane supramolecular chemistry has much in common with bottom-up synthetic biology, which seeks to build cellular systems with some of the functions of living cells (compartmentalisation, information processing, replication, chemical reactivity) from “simple” components. In synthetic biology, these components are typically large biomolecules such as proteins, nanopores, DNA *etc*. In supramolecular chemistry, these are transporters, channels, catalysts and artificial signal transducers. A key benefit of membrane confinement is that it enables the physical separation of different chemical environments – which may be chemically incompatible – on either side of the bilayer. This property has been used to create multi-compartment reaction vessels, such as in droplet interface bilayer systems, where the flow of material between the compartments is facilitated by protein nanopores and enables control over enzymatic reactivity and inter-compartment signalling.^176–181^ However, the transport selectivity of non-functionalised nanopores is comparatively poor, and cannot be controlled by external stimuli. Whilst it is possible to modify protein nanopores by building-in stimuli-responsive behaviour,^182^ it is interesting to consider whether synthetic supramolecular transport and signalling systems, particularly those that are stimuli-responsive and readily accessible, may find utility in complex artificial cells. We envisage that this may prove to be a fruitful area of collaboration between the fields of synthetic biology and supramolecular chemistry in the near future.

### Applications in biology and medicine

One of the key motivations for developing synthetic transport systems has been the promise of novel treatments for diseases arising from mis-regulated ion transport proteins (known as channelopathies), or in anti-cancer therapies. The field of “supramolecular medicinal chemistry”, with particular focus on applications of anionophores, has been reviewed extensively, and readers are directed to recent reviews.^71,73,183^ Before anionophores find their way from the lab to the clinic, there are arguably many hurdles to overcome, including optimising activity, ion selectivity, delivery and targeting, and toxicity. One of the often explored parameters in anionophore design is activity (typically the more active, the better), but as discussed previously, selectivity is also critical, because the presence of an anionophore will inevitably impact cellular homeostasis. In the case of potential anionophore-based therapies for cystic fibrosis, selective chloride transport in combination with low toxicity is desired, whilst anti-cancer therapies aim to induce tumour cell death.

Gale, Sheppard, Davis and co-workers have highlighted the real potential of anionophores as cystic fibrosis treatments by demonstrating that carriers, based on *trans*-decalin scaffolds bearing thiourea groups, mediate anion transport in yellow fluorescent protein (YFP)-modified cystic fibrosis airway epithelial cells.^184^ These experiments also showed that the anionophores were additive with the activity of known cystic fibrosis transmembrane regulator (CFTR) pontentiator ivacaftor and cystic fibrosis corrector lumacaftor, both of which are already used in the clinic. Similar results have also been reported by Moran and co-workers,^185^ thus demonstrating the potential of combined therapeutic strategies employing anionophores. This study also highlighted how a careful balance must be found between toxicity and anionophore activity.^184^ Indeed, whether an anionophore is toxic remains hard to predict, and it is not always directly related to its anion transport capability. However, there is growing evidence that the ability of certain anionophores to induce cell death is related to their anion/proton transport ability, and thus the ability to dissipate pH gradients.^71^ For example, the HCl transporter prodigiosin has been shown to rapidly de-acidify lysosomes, without compromising the membrane integrity, leading to a decrease in the cytosol pH and apoptotic cell death.^186^ We suggest that focus must now be directed to the challenge of mediating highly selective anion transport; for example chloride transport without competing proton/hydroxide transport for potential cystic fibrosis therapies. Some approaches towards this goal have been discussed earlier, including receptors with a high degree of encapsulation or use of sigma hole interactions. Much more progress is needed in this area, however, in particular with regards to developing transport motifs that are suitable for taking through the drug development process to the clinic.

Ion transporters are intrinsically highly lipophilic in order to effectively partition into the membrane. However, this poses a significant challenge for the efficient delivery of these highly lipophilic molecules to a target cell membrane. One approach to overcome this challenge has been reported by Kros and co-workers, who used liposomes as delivery vehicles for anionophores,^187^ but further work in this area is required. We anticipate that this may prove a fruitful area of collaboration between supramolecular chemistry and researchers working in the area of targeted drug delivery in the future. Reducing off-target effects is a crucial area of research in drug delivery, and this has inspired the development of stimuli-responsive transport systems that can be activated with spatio-temporal precision.^148^ We anticipate that this area will continue to grow, and we hope to see the rise of therapeutic transporters with targeted activity in the future.

## Conclusions

In this perspective we have discussed the current state of the art in supramolecular chemistry in lipid bilayer membranes. The field has expanded significantly in recent years, and is now a well-established sub-field of supramolecular chemistry, which is beginning to deliver new technologies applicable to biology and medicine. Confinement of supramolecular systems within the membrane of artificial vesicles has delivered a wide variety of functional systems spanning receptors, sensors, signal transducers, catalysts and transporters. Exerting spatio-temporal control over responsive systems promises to increase targeting and selectivity. So far, the vast majority of systems have been studied using relatively simple vesicles or planar lipid bilayers, but we are beginning to see much greater use of living cells, particularly in the emerging area of therapeutic anionophores. We anticipate that the ability to interface functional supramolecular systems with biology, by confining and solubilising the components within a membrane, will lead to many important applications within biology in the future, and firmly establish the field of supramolecular medicinal chemistry.

## Author contributions

L. E. B. wrote the section on mobile ion transporters, T. G. J. the section on siganl transuction and catalysis and A. K. the section on ion channels. M. J. L. conceived and directed the writing of the perspective, and wrote all other sections of the manuscript. All authors contributed to decisions on the overall content and the editing of the perspective.

## Conflicts of interest

There are no conflicts to declare.

## Supplementary Material
